# A Cryptic *CBFB* Deletion–Inversion Expands the Mutational Spectrum of Variants Associated With Cleidocranial Dysplasia

**DOI:** 10.1111/cge.14709

**Published:** 2025-02-02

**Authors:** Alistair T. Pagnamenta, Mona Hashim, Joanna Kennedy, Beth Lawton, Amaka C. Offiah, Jenny C. Taylor, Sarah F. Smithson

**Affiliations:** ^1^ Oxford BRC, Centre for Human Genetics University of Oxford Oxford UK; ^2^ Department of Clinical Genetics University Hospitals Bristol NHS Foundation Trust Bristol UK; ^3^ Division of Clinical Medicine The University of Sheffield Sheffield UK

**Keywords:** core‐binding factor complex, palindrome, skeletal dysplasia, structural variant

## Abstract

*CBFB* encodes the core‐binding factor β subunit, a small protein which heterodimerises with RUNX1‐3 and activates transcription of genes important in bone development. Recently, five families with cleidocranial dysplasia (CCD) were identified harbouring presumed loss of function variants in *CBFB*. Prompted by a multidisciplinary team review of an affected mother and daughter from the 100 000 Genomes Project with genetically unsolved CCD, we inspected read alignments and identified a deletion–inversion–deletion that removes the first two exons of *CBFB*. This cryptic variant comprised interlinked deletions of 1310 bp and 1935 bp and had remained undetected by both array‐CGH and the Canvas algorithm. The rearrangement was likely mediated by a palindromic AluSx repeat < 1 kb from the transcriptional start site. Due to high GC content and repeats, reduced read depth is observed at one of the breakpoints. Although the clinical presentation of *CBFB*‐related CCD appears to be very similar to *RUNX2*‐related CCD, our patients were of normal stature. The mild developmental delay observed in previously reported cases of *CBFB*‐related CCD was not observed. In conclusion, our data strengthens the evidence linking aberrations of the core‐binding factor complex to CCD and extends the mutational spectrum of pathogenic variants.

## Graphical abstract

Following clinical review of a 100 000 Genomes Project family with genetically‐unsolved CCD, we manually inspected read‐alignments and identified a deletion‐inversion‐deletion that removes the first two exons of *CBFB*. This cryptic variant comprised deletions of 1310/1935bp, was undetectable by array‐CGH and was likely mediated by palindromic AluSx repeats.

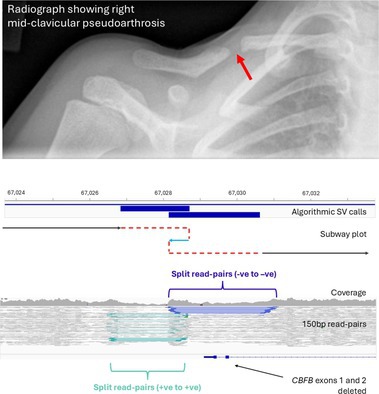

## 1 Introduction

Cleidocranial dysplasia (CCD) is characterised primarily by delayed closure of the cranial sutures and fontanelles, hypoplastic/absent clavicles and dental abnormalities. A study using the Utah Birth Defect Network estimated the prevalence to be ~1:80000 [1]. Rare variants in *RUNX2* underlie the majority of cases (CCD type 1; MIM #119600) and variant segregation is consistent with an autosomal dominant mode of inheritance. Between 10 and 30% of individuals with CCD cannot be explained by *RUNX2* variants [2, 3, 4].

*CBFB* encodes the 187 residue β subunit of the core‐binding factor complex (CBFβ) which forms a heterodimer with RUNX2 and regulates transcription of target genes critical for chondrocyte/osteoblast differentiation. Given this interaction, previous studies proposed *CBFB* to be a strong candidate gene for unsolved cases of CCD [5], a hypothesis supported by reports of 16q22 microdeletions (MIM #614541) in individuals with cranial features of CCD [6, 7].

Recently, Beyltjens et al., via GeneMatcher (https://genematcher.org), assembled eight individuals from five families with CCD harbouring presumed loss of function variants in *CBFB* (MIM #620099) [8]. In two families the variant arose *de novo*, whilst in three families the variant was maternally inherited. Further elucidation of rare *CBFB* genotypes/phenotypes will assist in diagnosis, especially given the intrafamilial clinical variability of CCD. Here, we describe a mother/daughter with *RUNX2*‐negative CCD and how manual review of whole genome sequencing (WGS) data identified a complex structural variant involving *CBFB*. The cryptic nature of this rearrangement was investigated by reviewing the genomic architecture and read coverage across gnomAD data.

## 2 Methods

The 100 000 Genomes Project (100kGP) is a UK‐wide study that aimed to demonstrate the utility of WGS for rare disease and cancer patients [9]. Ethics approval was from Cambridge South REC (14/EE/1112). This project has led to the widespread deployment of WGS across the NHS. In a process described previously [10], we have reviewed unsolved musculoskeletal cases from the 100kGP at monthly multidisciplinary team (MDT) meetings since February 2021. Data analyses, including library preparation and programmes used for variant detection, are described in Supporting Information.

Variant validation used three breakpoint PCRs and the FastStart PCR kit (Roche). GC‐rich additive was included. The first set of primers (5’‐CCAGATGACCGGACTTCAGA‐3′ and 5’‐CTTGAGGTCAGGAGGCCAG‐3′) captured both breakpoints. Primer set 2 (5’‐CCCTTGGTTGGCTTGAATCC‐3′ and 5’‐GATGGGTCTTAGATGGGATCC‐3′) captured just the proximal breakpoint, whilst set 3 (5’‐TGGAGAAAGTCTGACATGGGA‐3′ and 5’‐CTTGAGGTCAGGAGGCCAG‐3′) captured the distal breakpoint. Amplicons were size‐checked on a 1.8% agarose gel, purified using exonuclease I and Shrimp Alkaline Phosphatase followed by Sanger sequencing. Gene‐level variant coordinates and exon numbering are based on NM_022845.3(CBFB). Quantitative RT‐PCR methods are detailed in Supporting Information.

## 3 Case Report

**FIGURE 1:**
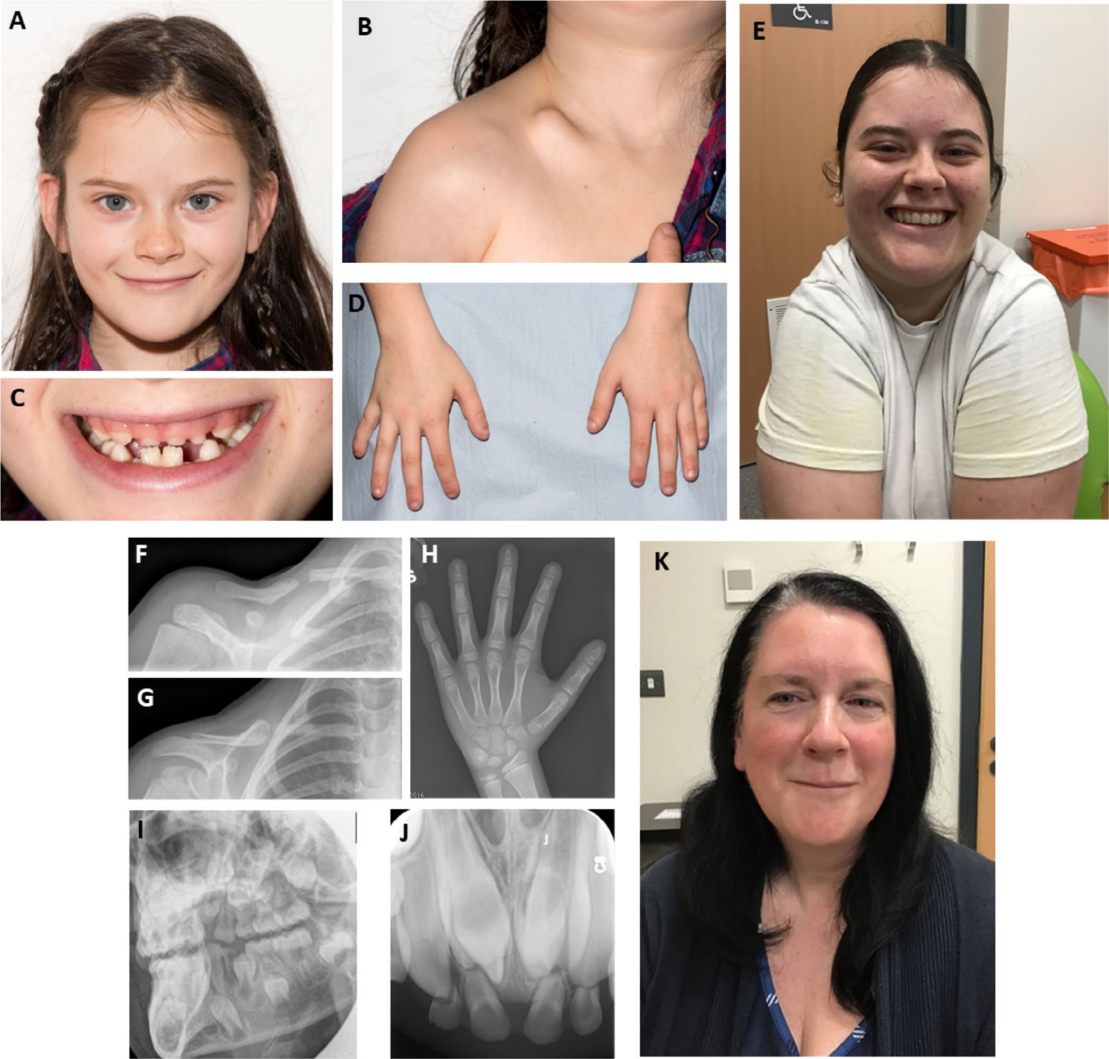
Images for family with CCD. (A) Proband at 9y without a marked facial difference, (B) right clavicular prominence at site of pseudoarthrosis, (C) irregular dentition, (D) short digits. (E) Marked shoulder approximation at 16y. (F,G) Anteroposterior and oblique radiographs of right clavicle at 5y showing mid‐clavicular pseudoarthrosis. (H) Anteroposterior radiograph of left hand at 8y shows a pseudoepiphysis of first metacarpal, hypoplastic terminal phalanges and acro‐osteolysis of terminal phalanges. (I,J) Lateral oblique of mandible and AP dental radiograph at 8y show supernumerary teeth. (K) Proband's mother also without facial signs of CCD.

The proband is a white British 16‐year‐old female, born following a normal pregnancy at 38/40 weeks' gestation by elective caesarean section and weighing 3.1 kg (50th centile). She initially presented at 8 weeks of age with a right clavicular fracture, later confirmed to be a pseudoarthrosis and a large anterior fontanelle. During early childhood, her motor development was slightly delayed and she was noted to have very mobile joints (skeletal features are presented in Figure 1A–J).

Although she had mild disproportionate short stature with a short spine during childhood, her final height is 163.4 cm (50th centile) and she weighs 68.2 kg (75th–91st centile). She has a full range of joint movements, no skeletal deformities and her spine is straight. The hands show bilateral shortening of the terminal phalanges with curved, symmetrical nails. Abnormal dentition (Figure 1C) included embedded supernumerary teeth complicated by abscesses which required extraction of eight primary teeth and gum surgery to allow her secondary incisors to descend: orthodontic treatment was successful. Skeletal survey confirmed the right clavicular pseudoarthrosis and small terminal phalanges of hands and feet but not major skeletal dysplasia. DEXA scans at 14 and 15y showed lumbar spine *Z* scores of −1.4 and −0.9 and sub‐total whole‐body scores of −1.6 and −1.2, respectively, within the normal range and improving with age.

Her affected mother (Figure 1K) had a wide fontanelle, supernumerary teeth requiring extraction and late eruption of adult teeth including impacted upper central incisors requiring dental intervention. She also experiences recurrent episodes of otitis media and has a mild scoliosis with degenerative changes in her spine. Her final adult height is 165 cm (50th centile) and her clavicles are clinically normal. The proband's sister and brother are unaffected. Previous genetic testing and primary analysis in the 100kGP are described in Supporting Information.

## 4 Results

**FIGURE 2:**
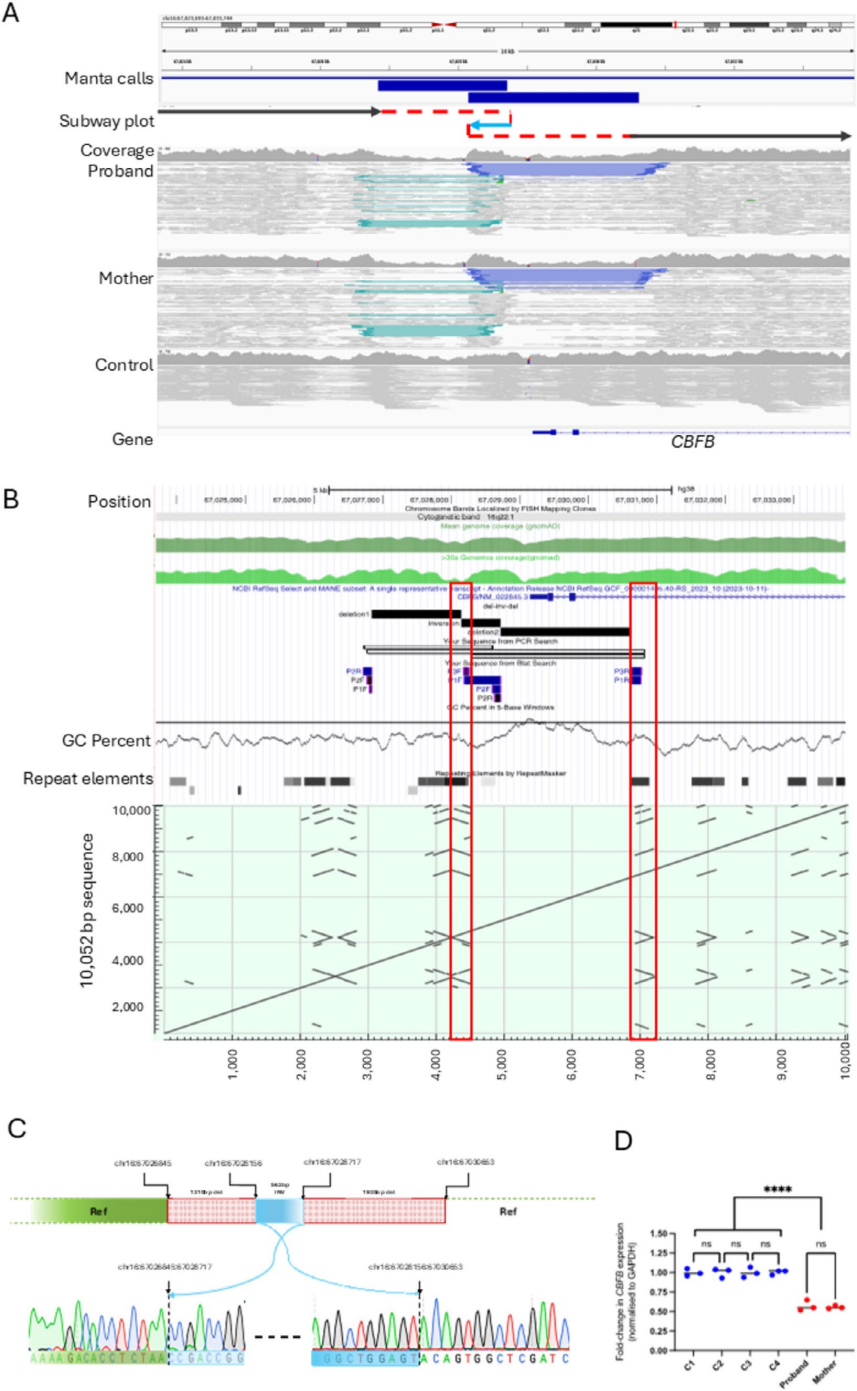
A cryptic structural variant disrupting *CBFB* confirms a clinical diagnosis of cleidocranial dysplasia. (A) IGV read‐alignments showing a DEL‐INV‐DEL that disrupts *CBFB* (NM_022845.3). The SV was called as two overlapping inversions by Manta but was not detected by Canvas. The region shown is chr16:67 023 693‐67 033 744 (GRCh38). Horizontal black and blue arrows in the subway plot indicate non‐inverted and inverted chromosome segments, respectively, whilst dotted red lines indicate junctions. (B) Primers used to validate the sequence and orientation of the SV are shown in the UCSC session: https://hgw1.soe.ucsc.edu/s/mhashim/CBFB_DEL_INV_DEL_Sanger. The dot plot (using the reference sequence for region shown in panel A) highlights regions with direct or inverted repeats. The red boxes indicate the presence of Alu elements and a palindrome‐like structure at the breakpoints of the SV. The horizontal line in the “GC‐content” track denotes an 80% threshold. (C) Sanger sequencing electropherograms confirm the inversion calls are part of the same complex SV. (D) Quantitative RT‐PCR results for *CBFB* normalised to *GAPDH* indicate that the proband and mother both have ~50% reduced expression compared to 4 controls. Representative results shown are using primers in exons 4/6.

An MDT‐based review of the clinical and radiological data substantiated the diagnosis of CCD. Radiological images at age 5 years showed abnormal clavicles (Figure 1F,G), hypoplastic terminal phalanges (Figure 1H) and supernumerary teeth (Figure 1I,J). Considering a recent study on CCD^8^, we undertook manual scrutiny of read‐alignments and identified a heterozygous *CBFB* deletion–inversion–deletion in the proband, shared by the mother (Figure 2A). Deletions of 1310 bp and 1935 bp flanked the central inverted segment of 562 bp. The larger deleted segment removes exons 1 and 2 of *CBFB*.

Although the variant was detected by manual review of read‐alignments, the SV had been called as two overlapping inversions by Manta (Figure 2A). However, the small size of the deleted segments and variable coverage due to high GC content meant that the deleted segments had not been detected by Canvas, with a 23.7 Mb reference call (chr16:46401886‐70 121 351) spanning this region (Supporting Information Note 1). Using the larger of the inversion calls, we searched the SVRare database that includes aggregated data for 71 408 individuals from the 100kGP [11] and this indicated the SV to be private to this family.

Based on data from gnomAD, we estimated that only 3.5% of WGS datasets had coverage of 30x or more at the proximal end of the inverted segment. This low‐coverage region harbours an inverted pair of AluS elements constituting a palindrome‐like sequence (Figure 2B). Using three overlapping PCRs, we validated the SV using breakpoint PCR and Sanger sequencing (Figure 2C). This resolved the precise genomic coordinates of the rearrangement to be NC_000016.10:g. 67026846_67030652delins67028156_67028717inv. Quantitative RT‐PCR suggests that the structural variant results in significantly reduced levels of *CBFB* transcript (Figure 2D).

Using the ACMG guidelines [12], this SV was assessed as pathogenic based on PM2, PP4 and PVS1. PVS1 is supported by the removal of both transcriptional and translational start sites. As the next methionine codon is not until p.Met101, this would be unlikely to confer any alternate start‐site rescue effect.

## 5 Discussion

A recent study assembled five unrelated families with *RUNX2*‐negative CCD harbouring variants in *CBFB* [8]. This set of reported variants predominantly had severe consequence predictions, including two frameshifts (p.Cys25Tyrfs*2 and p.Pro100Leufs*3), one stop‐gain (p.Arg83*) and a 9003 bp deletion of exon 4 which predicts in‐frame deletion of (p.Val95_Gln133del). Another microdeletion was previously estimated at 1.2 Mb in size [6]. The deletion–inversion–deletion described here represents the first complex SV to be reported involving *CBFB*. Based on the five previously described *CBFB* families and our replication in 100kGP, the PanelApp status for this gene (https://panelapp.genomicsengland.co.uk/panels/309/gene/CBFB) has been updated to green.

Palindrome‐like structures in the genome can mediate the formation of recurrent complex SVs and examples of this phenomenon in the literature include rearrangements involving *SRRM2* [13], *NF1* [14] and *SOX3* [15]. Given the abundance of Alu elements and palindrome structure upstream of *CBFB* and literature regarding recurrent inversions linked to laeukaemia (Supporting Information Note 2), the possibility of cryptic germline rearrangements should be strongly considered when assessing future cases of genetically unsolved CCD. Given the small size of the gene, manual review of alignments is relatively straightforward, if appropriate IGV settings are employed.

The two affected family members described here showed significant phenotypic differences as well as similarities. Whilst both exhibited supernumerary teeth and large fontanelles, the affected mother has scoliosis but not the clinical clavicular anomalies. Such intra‐familial heterogeneity is a common theme in CCD. Given this variability, comparisons between CCD types 1 and 2 would ideally involve larger groups of patients. However, Beyltjens et al. noted that 3/8 patients in their *CBFB*‐related CCD cohort had developmental delay, which is not a feature of CCD patients with *RUNX2* variants [8]. It is speculated that this could be because CBFβ interacts with other RUNX proteins. However, no developmental delay was seen in the present family so confirmation of this additional phenotype association would require further support. Other aspects of the *CBFB*‐related CCD phenotype appear to be milder. Unlike patients with *RUNX2*‐related CCD, those with *CBFB* variants appear to have a normal pelvis and as was observed here, in most cases are of normal stature.

There are also phenotypic differences apparent between individuals with 16q22 microdeletion syndrome and the *CBFB*‐related CCD families [8]. For the published microdeletion cases, dental/clavicular anomalies were not reported. Family 2 in the cohort of Beyltjens et al. harboured a 9003 bp deletion that removes exon 4 and RNA studies showed that the reading frame remained intact [8]. RNA studies on samples from Family 1 also showed no evidence of NMD and this led to speculation about possible neomorphic effects due to expression of a truncated protein. Our data argues against that hypothesis as the SV removes both transcriptional and translational start sites and qPCR data showed a significant reduction in transcript levels.

In conclusion, manual scrutiny of genomic data identified a cryptic SV that extends the mutational spectrum of variants linked to *CBFB*‐related CCD. Additional studies further delineating these clinically heterogeneous syndromes will lead to a better understanding of genotype–phenotype correlations. Assessment of how deficits in core‐binding factor influence downstream gene regulation and result in deficits in skeletal development may in the future lead to the development of therapeutic options.

## Author Contributions

A.T.P., J.C.T. and S.F.S. conceived the project. A.T.P. and B.L. performed data analysis. B.L. and M.H. generated/interpreted genetic validation data. S.F.S. recruited the family and with J.K. collected clinical data. A.O. reviewed radiographs. A.T.P. drafted the manuscript with all co‐authors.

## Conflicts of Interest

The authors declare no conflicts of interest.

## Peer Review

The peer review history for this article is available at https://www.webofscience.com/api/gateway/wos/peer‐review/10.1111/cge.14709.

## Supporting information


**Data S1.** Supporting Information.

## Data Availability

100kGP data are in the National Genomic Research Library (https://doi.org/10.6084/m9.figshare.4530893.v6).
