# Supplementary material for: A Cryptic CBFB Deletion–Inversion Expands the Mutational Spectrum of Variants Associated With Cleidocranial Dysplasia
Source: Clin Genet. 2025 Feb 2;108(1):86–91. doi: 10.1111/cge.14709 (PMC12136864; doi:10.1111/cge.14709)
Supplement: Supplementary file 1 — Data S1. Supporting Information. [file CGE-108-86-s001.docx]

**Supplementary material: A cryptic *CBFB* deletion-inversion expands the mutational spectrum of variants associated with cleidocranial dysplasia**

**Supplementary Methods**

***Genome sequencing and data analysis***

Library preparation was using TruSeq PCR-Free High Throughput kit and blood collected in EDTA tubes. WGS was performed on a HiSeqX as part of the 100kGP, using a 150bp paired-end format (Illumina). One sample was run per lane and data was processed on the Illumina North Star WGS Workflow (NSV4, version 2.6.53.23); which comprises the iSAAC Aligner (version 03.16.02.19) and Starling Small Variant Caller (version 2.4.7). Samples were aligned to the *Homo Sapiens* GRCh38 assembly, with decoys. Detection of structural variants (SVs) was using Manta and Canvas algorithms and allele frequencies were determined from aggregated data for 71,408 individuals using SVRare, as described.^1^

Read-alignments were viewed using IGV (version 2.15.4) and the “view-as-pairs” option. Alignments were coloured by “insert-size and pair orientation” and sorted by insert size. WGS coverage statistics were generated from gnomAD v2.1.1, with genomic coordinates converted to GRCh38 using the LiftOver tool (<https://genome.ucsc.edu/cgi-bin/hgLiftOver>). Dot plots were created using the Nucleotide BLAST tool (<https://blast.ncbi.nlm.nih.gov/Blast.cgi>) with the ”align two or more sequences” and “somewhat similar sequences” options.

***RNA analysis***

RNA was extracted from Tempus blood RNA tubes using the Preserved Blood RNA Purification Kit I (Norgen Biotek). Concentration and purity were measured using the NanoDrop. The 260/230 ratio for both samples was between 2.00 and 2.13 and the 260/280 ratio was between 2.00 and 2.02, indicating pure RNA. Concentrations were 548.9ng/µl for the proband and 623.5ng/μl for the mother. cDNA Synthesis was performed using ~1µg of RNA as input and the QuantiTect Reverse Transcription Kit (Qiagen). To remove genomic DNA contamination, RNA samples were first mixed with gDNA Wipeout Buffer (1000ng template RNA and 2μl gDNA Wipeout Buffer with RNase-free water to make up 14μl) and incubated at 42°C in the thermocycler for 2 minutes. Next, samples were mixed with a mastermix consisting of reverse transcriptase and primers (1μl QuantiTect Reverse Transcriptase, 1μl RT Primer Mix and 4μl QuantiTect RT Buffer). The thermocycler protocol was continued at 42°C for 30 minutes to generate cDNA and 95°C for 3 minutes to inactivate the reverse transcriptase. Negative control samples were included, replacing QuantiTect Reverse Transcriptase with RNase-free water (RT-ve), to identify possible genomic DNA contamination. PCR reactions were performed on the CFX96 Touch Real-Time PCR Detection System using iQ SYBR Green Supermix (Bio-Rad). Reaction volumes were 20μl and ~25ng of cDNA was used as template. Reactions were set up in triplicate, with two runs from independent RNA extractions (we were supplied with two Tempus tubes for both the mother and the proband). Four healthy adult female controls were also used and normalisation was to either the *HPRT1* or *GAPDH* housekeeping genes. Primer sequences, amplicon sizes and exons targeted are shown in Table S1 whilst cycling conditions are shown in Table S2.

***Previous genetic testing and primary analysis in the 100kGP***

Previous genetic testing had included di-deoxy sequencing of the coding sequence and exon-intron boundaries of *RUNX2* (NM_001024630.4). Copy Number Variant (CNV) analysis was also undertaken in 2016 using the 8x60k ISCA v2.0 array-CGH platform (Oxford Gene Technology, average resolution 180kb) and CytoSure Interpret version 4.7 software. Targeted CNV analysis used a multiplex ligation-dependent probe amplification kit, examining 10 genes linked to craniofacial disorders (P080-C1, MRC Holland) and this included 9 probes for *RUNX2*. None of these approaches detected any clinically relevant abnormalities and so the proband and her mother were recruited to the 100kGP in April 2017 and analysed as a duo. The primary analysis pipeline used in the 100kGP for this family used the *in silico* panels for thoracic dystrophies (<https://panelapp.genomicsengland.co.uk/panels/122/>, version 1.1) and skeletal dysplasia (<https://panelapp.genomicsengland.co.uk/panels/309/>, version 1.89). This did not uncover any plausible diagnostic variants and the case was signed off as unsolved (pending future reanalysis) in December 2019.

**Table S1:** Primer sequences for quantitative RT-PCR reactions. For *CBFB*, amplicon size and exon numbering is based on transcript NM_022845.3. Housekeeping genes used as controls were *GAPDH* (NM_002046.7) and *HPRT1* (NM_000194.3).

| **Name** | **Primer sequence** | **Amplicon size** | **Exons** |
| --- | --- | --- | --- |
| *CBFB* set 1 | 5’-AGCAAGTTCGAGAACGAGGAGT-3’  5’-CAAAAGCGATTTCCGAGCGG-3’ | 145bp | 1-3 |
| *CBFB* set 2 | 5’-AACAGCGACAAACACCTAGC-3’  5’-CCAGCCTTTCCAGATAACACAG-3’ | 113bp | 3-4 |
| *CBFB* set 3 | 5’-TTGATGAGGAGCGAGCCCAG-3’  5’-ACCAGGACTAGGGTCTTGTTGTC-3’ | 146bp | 4-6 |
| *GAPDH* (control) | 5’-GCACCACCAACTGCTTAGC-3’  5’-GTCTTCTGGGTGGCAGTGAT-3’ | 106bp | 7-8 |
| *HPRT1* (control) | 5’-TGACACTGGCAAAACAATGCA-3’  5’-GGTCCTTTTCACCAGCAAGCT-3’ | 94bp | 6-7 |

**Table S2:** Quantitative RT-PCR cycling conditions.

| **Cycling step** | **Temperature** | **Hold time (min:sec)** | **# of cycles** |
| --- | --- | --- | --- |
| Initial denaturation and enzyme activation | 95°C | 2:00-3:00 | 1 |
| Denaturing | 95°C | 0:10-0:15 | 39 |
| Annealing and extension | 55-60°C | 0:30 |  |
| Melt curve | 55-95°C (in 0.5°C increments) | 0:10-0:30 | 1 |

**Supplementary Notes**

**Supplementary Note 1**

In the human genome, high GC content is often observed in the promoter and 5’-UTR exon 1 regions of genes. This can often result in reduced read coverage in both exome and genome sequencing datasets. In the case described here, the breakpoints did not lie in coding sequence and given the variable coverage, the SV would likely have been missed by exome analysis. Even with genome sequencing data, the deletions had remained undetected by CANVAS algorithm. We previously described a similarly cryptic 3kb deletion in the GC-rich region of *ARX* exon 1, which required MLPA testing to validate.^2^

**Supplementary Note 2**

*CBFB* is also known to geneticists through its link to acute myeloid leukaemia.^3^ A relatively common pericentric inversion inv(16)(p13q22) leads to a *CBFB-MYH11* fusion-gene which is thought to confer disease susceptibility via a gain-of-function mechanism.^4^ These somatic *CBFB* breakpoints are typically found in intron 5, whereas the germline deletion-inversion-deletion reported in the current study removes exons 1-2 and thus removes both the canonical transcriptional and translational start sites.

**References**

1. Yu, J., Szabo, A., Pagnamenta, A.T., Shalaby, A., Giacopuzzi, E., Taylor, J., Shears, D., Pontikos, N., Wright, G., Michaelides, M., et al. (2022). SVRare: discovering disease-causing structural variants in the 100K Genomes Project. medRxiv, 2021.2010.2015.21265069. 10.1101/2021.10.15.21265069.

2. Pagnamenta, A.T., Camps, C., Giacopuzzi, E., Taylor, J.M., Hashim, M., Calpena, E., Kaisaki, P.J., Hashimoto, A., Yu, J., Sanders, E., et al. (2023). Structural and non-coding variants increase the diagnostic yield of clinical whole genome sequencing for rare diseases. Genome Med *15*, 94. 10.1186/s13073-023-01240-0.

3. Liu, P.P., Hajra, A., Wijmenga, C., and Collins, F.S. (1995). Molecular pathogenesis of the chromosome 16 inversion in the M4Eo subtype of acute myeloid leukemia. Blood *85*, 2289-2302.

4. van der Reijden, B.A., Dauwerse, H.G., Giles, R.H., Jagmohan-Changur, S., Wijmenga, C., Liu, P.P., Smit, B., Wessels, H.W., Beverstock, G.C., Jotterand-Bellomo, M., et al. (1999). Genomic acute myeloid leukemia-associated inv(16)(p13q22) breakpoints are tightly clustered. Oncogene *18*, 543-550. 10.1038/sj.onc.1202321.
